# State Control of Glanders in Minnesota1Read before the American Veterinary Medical Association, New York, September, 1899.

**Published:** 1900-01

**Authors:** M. H. Reynolds

**Affiliations:** St. Anthony Park, Minn.


### STATE CONTROL OF GLANDERS IN MINNESOTA.[1]

By M. H. Reynolds, D.V.M.,
ST. ANTHONY PARK, MINN.

(Concluded from page 742, December number, 1899.)

Our glanders placard when signed and posted constitutes in most cases the official written order of quarantine. It reads as follows:

Glanders Exists upon these Premises.

All persons are forbidden to remove from these premises, without written permission from the State or Local Board of Health, any animal or thing whereby glanders may be conveyed.

No quarantined horse or mule may under any circumstances be placed in any other stable; or tied to any public hitching-post or rack; or be watered in or from any trough or vessel to which other horses or mules have access.

No horse or mule, except those hereby quarantined, may under any circumstances be allowed in these stables or in any stables that have been quarantined on these premises.

The owner or keeper of these premises will be held responsible for the unauthorized removal of this card.

By order of

——— ———
Health Officer.

Dated ———, 18—.

Chapter 233, Laws of 1897.

Section 1. Authority is hereby given to the State Board of Health and to the several local boards of health of the towns, villages, and cities of this State to take all steps they may severally deem necessary to control, suppress and eradicate any and all contagious and infectious diseases among any of the domestic animals in this State, and to that end said boards are hereby severally empowered, within their respective jurisdictions, to quarantine any domestic animal which is infected with any such disease or which has been exposed to infection therefrom.

Section 9. Any person violating any provision of this act, or any rule

[1] Read before the American Veterinary Medical Association, New York, September, 1899.

or regulation made by the State Board of Health, or by any Local Board of Health, or any order made by any such board under the authority hereof, shall be guilty of a misdemeanor and be punished by a fine of not less than twenty-five (25) or more than one hundred (100) dollars, or by imprisonment for not less thirty (30) or more than ninety (90) days.

The following is our "Notice to Destroy."

(This part to be retained by the local board.)

Always fill out as early as possible, for each outbreak of glanders, one of the blanks for reporting infectious diseases among animals and return to Dr. M. H. Reynolds, St. Anthony Park, Minn.

NOTICE TO DESTROY ANIMALS HAVING GLANDERS–FARCY.

189 Given by local Board of . . . Notice served by . . . Owner's or keeper's name and address. . . . Name and description of animal.

(For the owner.)

189 . . . To . . . You are hereby notified that there is now on your premises the animal (give name, age, color, etc.) . . . suffering from glanders-farcy, which disease is contagious to man and animals. You are hereby ordered to have said animal killed within twenty-four hours after the service of this notice and to have the stables and things occupied and used by said animal disinfected in accordance with the following regulations:

REGULATIONS FOR DEALING WITH GLANDERS-FARCY.

By authority of law, Chapter 233, approved March 12, 1897.

The slaughter of condemned animals, their burial and disinfection of stables, etc., as herein prescribed, must be done under the supervision of the health officer or chairman of local board.

When any local board of health has satisfactory evidence that any horses or mules are affected with glanders-farcy, such horses or mules must be killed within twenty-four hours ot the service of this notice.

Each carcass must be covered with quicklime before filling in any earth, and each carcass, after slashing the hide, shall be buried so that the entire body shall be at least four feet below the surface of the ground.

No sales to rendering establishments or other disposition of the carcass than as above provided shall be permitted.

Parties who attend the killing and burial should be warned that the disease may be readily contracted by them and the disease is very fatal to human beings. Such infection may occur through the mucous membranes of the eyes, nose, or mouth, or through any cut or abrasion of the skin.

After disposing of the carcass, the next step is cleaning and disinfection of the stable and articles used by the condemned animals.

Remove and burn all litter, including hay in the mangers, and bedding.

Scrape the floor clean as possible, and burn the manure and dirt that may be scraped from the floor.

Close the stable tightly as possible, filling up the holes and cracks; place in the stable from one to five washtubs of boiling water, the number to be used depending on the size of stable. On stones or bricks in each tub of water place an iron kettle or old pan; then burn in each pan or kettle three pounds of ordinary sulphur that has been saturated with alcohol. All persons should go out as soon as possible and shut the door tightly, the stable to remain tightly closed for ten or twelve hours; then open up and ventilate thoroughly for twenty-four hours.

Everything that cannot be boiled should be left hanging in the stable or burned; and when the fumigation is finished, such articles should be hung out in the open air and exposed to sunshine for several days.

The interior of the stable should be whitewashed with fresh whitewash, containing one-fourth of a pound of chloride of lime per gallon, and lime should be scattered freely over the floors.

The interior of the mangers and feed-boxes must be thoroughly disinfected by boiling water or by corrosive sublimate solution, fifteen grains to the pint of water.

No horses or mules may be allowed in such stables until after at least a week of continuous exposure to free ventilation and sunshine following the above prescribed disinfection.

Temporary structures, like straw sheds, etc., should be burned, and the same is true of blankets and halters that have been used by diseased animals.

By order of the local board of health. ——— ———

Health Officer or Chairman.

CHAPTER 233—S. F. NO. 322.

*An Act to Prevent the Spread of Contagious and Infectious Diseases Among Domestic Animals in This State.*

Be it enacted by the Legislature of the State of Minnesota:

SECTION 1. Authority is hereby given to the State Board of Health and to the several local boards of health of the towns, villages, and cities of this State, to take all steps they may severally deem necessary to control, suppress, and eradicate any and all contagious and infectious diseases among any of the domestic animals in this State, and to that end said boards are hereby severally empowered, within their respective jurisdictions, to quarantine any domestic animal which is infected with any such disease or which has been exposed to infection therefrom; to kill any animal so infected, and whenever deemed necessary by the State Board of Health to kill any animal which has been exposed to the infection of any such disease.

SEC. 6. When any animal is quarantined upon the premises of its owner or keeper, the expense thereof shall be borne by its owner or keeper. Whenever any animal is quarantined when being shipped into the State, the expense thereof shall be borne by its owner or keeper. Whenever the owner or keeper of any domestic animal is liable for any expenses incurred, under this act, by any board of health in connection therewith, such board may have a lien on such animal for such expense, and may also maintain an action against such owner or keeper therefor.

SEC. 7. It is hereby made the duty of the several local boards of health in this State to carry out and enforce all orders and directions of the State Board of Health to them directed, and the State Board of Health may require any two or more local boards to act together for the purpose of enforcing any of the provisions of this act.

SEC. 9. Any person violating any provision of this act or any rule or regulation made by the State Board of Health, or by any local board of health, or any order made by any such board under the authority hereof, shall be guilty of a misdemeanor and be punished by a fine of not less than twenty-five (25) or more than one hundred (100) dollars, or by imprisonment for not less than thirty (30) or more than ninety (90) days. Any member of any local board of health who shall neglect or refuse to carry into effect the provisions of this act, or who shall neglect or refuse to carry out any directions of the State Board of Health, or who shall neglect or refuse to enforce any rule or regulation made by the State Board of Health, or by any local board of health, under the authority hereof shall be guilty of a misdemeanor and be punished by a fine of not less than twenty-five (25) and not more than one hundred (100) dollars; and each and every day's neglect or refusal to perform any duty imposed upon him by this act shall constitute a separate and independent misdemeanor. Complaints for violating the provisions of this act, or for violating any rule or regulation made by any board of health under its authority, may be made by any member or authorized agent of any such board or by any citizen of this State.

This circular was written two years ago, and will be revised in the next issue.

We also have a circular, "Notice to Isolate." This is served only in rare cases.

Results: It is perhaps not unfair that I should submit some of our records showing the results of these methods. Your attention is called to the marked improvement made in one and two years in the following counties selected as the ones in which glanders-farcy was most common during 1897. Those containing the large cities are omitted.

| | 1897. | 1898. | 1899. |
|---|---|---|---|
| Polk | 32 | 16 | 5 |
| Carver | 30 | 2 | 0 |
| Chippewa | 22 | 4 | 0 |
| McLeod | 11 | 0 | 0 |
| Winona | 8 | 0 | 1 |
| Kandiyohi | 8 | 0 | 3 |
| Redwood | 5 | 0 | 0 |
| Faribault | 7 | 2 | 1 |

Please bear in mind that our work is done without the substantial aid which comes from partial or complete compensation. These figures must show plainly to any one familiar with the peculiarities of this disease, and especially to those familiar with public works of this kind, that the work of 1897 was successful.

*General Problems.* The question, what constitutes a mallein reaction, certainly deserves consideration, inasmuch as there is difference of opinion among practitioners, particularly as to the basis for comparison of temperatures. A gradual rise of two degrees or more above the normal range within twenty-four hours, and a well-marked, painful swelling at the site of injection constitute a reaction upon which it is safe to diagnose glanders, providing other possible causes of these disturbances are borne in mind. The temperature reaction is entitled to much greater consideration than the local swelling. Horses affected with glanders-farcy occasionally give marked temperature reactions with very little local disturbance. A record of this kind should constitute a mallein reaction when occurring in a stable where an undoubted case of glanders-farcy had recently appeared. If there had been no such case of glanders in the stable, and the horses showed no clinical symptoms, I should hesitate before making positive diagnosis. Under ordinary circumstances we regard such an animal as suspicious, and the owner is forbidden to dispose of it for removal from the place for a reasonable time.

*What Horses should be Killed?* All that show plain symptoms of glanders-farcy, regardless of mallein reaction, and all horses that have given clear reactions and show any external symptoms of the disease, including thick wind and chronic cough.

What should be done with the horse that reacts but shows no other symptom of the disease? It is not necessary that all reacting horses should be immediately destroyed, but I do insist that we should know where the mild and latent cases of glanders-farcy are. We should have on file records concerning such cases. These horses should at least be kept under observation and should never be released until they have failed to react on proper mallein-test. I believe the Minnesota rule upon this point is a wise one. It is in effect that such horses may either be destroyed at once, or continued in quarantine for any period not shorter than thirty days nor longer than one year from date of test. Such horses cannot be released from quarantine until they have failed to react under mallein-test. If such horses develop clinical symptoms during quarantine they must be destroyed without delay. Beyond these points each case is decided upon its own merits. In many cases the full quarantine period is permitted and the owner is authorized to use one or more teams upon the road, according to circumstances. In the country farmers are usually authorized to use any of their quarantined horses in farm work, but they are isolated from healthy horses so far as stable, water, food, and work are concerned. Please bear in mind that horses which show clinical symptoms are not quarantined.

Protection for the honest owner offers another problem. Partial or full compensation cannot alone solve it. Conditions of quarantine may sometimes be modified so as to lighten his burden a great deal. When this is done we still have left for consideration the loss of property. Generous compensation is undoubtedly the theoretical solution, but sanitary authorities in comparatively few States are able to do this. It is sometimes difficult to protect the honest owner and the State. Compensation should be based upon appraisal, and appraisal should be limited. The State should not be compelled to pay any unreasonable prices for diseased animals. Neighbors are apt to be generous when the State is to pay the bill. Appraisal should not be permitted unless the condemned animal has been owned in the State at least six months and in possession of the present owner for at least sixty days. Neither should compensation be permitted if the animal was obviously sick at the time of purchase.

As the Minnesota law dealing with infectious diseases of animals now stands, we are not authorized to reimburse owners for animals that may be destroyed on account of infectious diseases. Our law provides that after an animal has been condemned for destruction the owner shall have twenty-four hours in which to make formal protest against the killing. Post-mortem is then conducted by three experts, and if these experts find no evidence of any infectious disease, then the animal may be appraised. The State pays four-fifths and the local board one-fifth. I do not consider this an ideal method. It is the best we can do at present. But we make a special effort to protect the owner by usually killing only those horses which show clinical symptoms.

I have been trying to devise some plan of optional compensation, so that those who really deserve help may have it without asking the State to pay for all animals that may be condemned. But it is difficult to discriminate in public work. The most feasible plan that has so far suggested itself is to ask for a law which shall provide that whenever the township supervisors or the city authorities first offer to pay one-third or one-half of the appraised sum then the State Board of Health shall pay the remainder. In this way leaving compensation optional with the local authorities who are familiar with the circumstances of the case, and at the same time testing their sincerity by asking them to help pay the bill.

How to secure prompt reports concerning the appearance of glanders-farcy is quite a problem. The Minnesota solutions is given in section 2 of the law, which reads as follows:

Sec. 3. Any person who knows of, or has reason to suspect, the existence of any contagious or infectious diseases in any domestic animal shall forthwith give notice thereof to the local board of health of the town, village, or city where such animal is kept. Within twenty-four (24) hours after any local board shall receive notice that any domestic animal is infected with any such disease, or has been exposed thereto, it shall give notice thereof in writing to the State Board of Health.

Disobedience is punishable by fine or imprisonment.

Practical disinfection offers another problem, but not so difficult. Löffler and others have demonstrated that the glanders bacillus is very sensitive to unfavorable conditions, that these germs are destroyed by ten-minutes' exposure to a temperature of 56° C., five minutes in 3 per cent. carbolic acid, three minutes in 1 : 5000 bichloride, that they fail to grow after a short time, even under most favorable conditions. These germs are very susceptible to light and probably to low temperatures. So that if we are able to keep a stable vacant for a comparatively short time, and especially if it be well lighted and ventilated, nature will do the disinfecting. Or we can whitewash, using a spray pump for distributing the whitewash. This can be followed by two or three men using brushes on the woodwork. For whitewashing walls, roofs, partitions, etc., the spray pump is efficient and very rapid. It may be that we will soon be able to use formaldehyde in a practical way. Capable experimenters who have worked with this agent have arrived at such different results that we naturally feel inclined to wait.

*The Large City.* Glanders-farcy in large cities offers a difficult problem. The theoretical solution is not at all difficult, but the practical solution is a somewhat different matter. All who have had experience will agree with me that it is much easier to do thorough and effective work in the country and in small cities. Rules may be made which are wise and practical for the country and correct in theory for the city, but which are extremely difficult of enforcement in the latter places; for instance, testing all horses that have been exposed in the stable. This may mean the testing of 50 or 100 horses, or possibly 250 horses, with a startling percentage of reactions. In the country we may allow the owner to use his team until after harvest, or use the team upon the road, or even to go to town under certain conditions. The risk of spreading the disease in this way is insignificant, but this is not true in the city. Public watering places are scattered everywhere. Places where horses are tied are abundant, and while the diseased horse may not be tied to any of these places, he is liable to be left standing where he can conveniently rub his nose. A diseased horse in the city exposes several times as many horses as a similar case in the country.

In the city we have the large livery-stable to deal with. An outbreak of glanders-farcy in a large livery-stable is a difficult thing to manage, if one tries to bear in mind the two fundamental propositions which I mentioned at the beginning of this paper. If we do not test all horses in the stable we are sure to miss some which are affected and which may develop into infectious cases within a short time. If we do test all horses in the stable and a number of horses react without showing symptoms of the disease then the difficult problem arises immediately: What shall we do with them? We cannot quarantine them without quarantining the stable. If we quarantine the stable and post the glanders placard it may mean ruin for the owner's business. We cannot trust hack- and cab-drivers to refrain from watering their horses at any public watering-place, from tying at or allowing them to stand near public hitching-places.

The public watering-place is another city problem, but not a difficult one. Small tanks and water rapidly changed is the evident solution.

These are but a few of the many problems which the large cities offer for solution. I could not discuss these problems fully in a paper of reasonable length, but would very much like to hear them discussed at this meeting. Some have been solved. Some are in the progress of solution.

---

The Christmas examinations of the Ontario Veterinary College, Toronto, Canada, were held in the Veterinary College, on Thursday, December 21, 1899.

The Board of Examiners, which is composed of prominent veterinary surgeons in the active practice of their profession in various parts of the country, after subjecting the candidates to a rigorous examination awarded diplomas to the following gentlemen: Alva G. King, Moosup, Conn.; Arthur N. Norwood, Naugatuck, Conn.; Millage Philips, Wallaceburg, Ont.; Wm. S. Shultze, Marengo, Iowa; David J. Smith, Barre, Vermont.

Dr. Charles F. Dawson, of the Bureau of Animal Industry, has resigned his chair as lecturer on pathology in the United States College of Veterinary Surgeons, Washington, D. C.

